# Cerebellar stimulation in schizophrenia: A systematic review of the evidence and an overview of the methods

**DOI:** 10.3389/fpsyt.2022.1069488

**Published:** 2022-12-22

**Authors:** Jessica P. Y. Hua, Samantha V. Abram, Judith M. Ford

**Affiliations:** ^1^Sierra Pacific Mental Illness Research Education and Clinical Centers, San Francisco Veterans Affairs Medical Center, University of California, San Francisco, San Francisco, CA, United States; ^2^San Francisco Veterans Affairs Medical Center, San Francisco, CA, United States; ^3^Department of Psychiatry and Behavioral Sciences, University of California, San Francisco, San Francisco, CA, United States

**Keywords:** transcranial stimulation, cerebellar vermis, schizophrenia, negative symptoms, depression, tDCS, TMS

## Abstract

**Background:**

Cerebellar structural and functional abnormalities underlie widespread deficits in clinical, cognitive, and motor functioning that are observed in schizophrenia. Consequently, the cerebellum is a promising target for novel schizophrenia treatments. Here we conducted an updated systematic review examining the literature on cerebellar stimulation efficacy and tolerability for mitigating symptoms of schizophrenia. We discuss the purported mechanisms of cerebellar stimulation, current methods for implementing stimulation, and future directions of cerebellar stimulation for intervention development with this population.

**Methods:**

Two independent authors identified 20 published studies (7 randomized controlled trials, 7 open-label studies, 1 pilot study, 4 case reports, 1 preclinical study) that describe the effects of cerebellar circuitry modulation in patients with schizophrenia or animal models of psychosis. Published studies up to October 11, 2022 were identified from a search within PubMed, Scopus, and PsycInfo.

**Results:**

Most studies stimulating the cerebellum used transcranial magnetic stimulation or transcranial direct-current stimulation, specifically targeting the cerebellar vermis/midline. Accounting for levels of methodological rigor across studies, these studies detected post-cerebellar modulation in schizophrenia as indicated by the alleviation of certain clinical symptoms (mainly negative and depressive symptoms), as well as increased frontal-cerebellar connectivity and augmentation of canonical neuro-oscillations known to be abnormal in schizophrenia. In contrast to a prior review, we did not find consistent evidence for cognitive improvements following cerebellar modulation stimulation. Modern cerebellar stimulation methods appear tolerable for individuals with schizophrenia, with only mild and temporary side effects.

**Conclusion:**

Cerebellar stimulation is a promising intervention for individuals with schizophrenia that may be more relevant to some symptom domains than others. Initial results highlight the need for continued research using more methodologically rigorous designs, such as additional longitudinal and randomized controlled trials.

**Systematic review registration:**

[https://www.crd.york.ac.uk/prospero/], identifier [CRD42022346667].

## 1 Introduction

The cerebellum was traditionally considered a primary driver of motor coordination (1); however, more current views acknowledge the cerebellum’s central role in multiple motor, cognitive, and behavioral functions (2–6). Indeed, it has been called a scholar and an athlete (7). Schizophrenia is characterized by psychotic symptoms, cognitive difficulties, and impairment in coordinated motor functioning and sensory processing. Converging evidence points to robust cerebellar abnormalities in schizophrenia that may impact an array of clinical symptoms, cognition, and behavior (8–10) likely because of the cerebellum’s widespread connections within the cortex (8, 10). The cerebellum is therefore a promising target for novel intervention development (11–13). Cerebellar brain stimulation methods are posited to modulate the cerebellum as well as distributed neural systems connected to the cerebellum (14, 15); this feature is particularly important in the context of schizophrenia and its conceptualization as a disorder of widespread dysconnectivity (16). In the current systematic review, we examine the potential of cerebellar stimulation as a treatment for schizophrenia and its associated symptoms.

### 1.1 Historical approaches for cerebellar stimulation

Prior to the current use of non-invasive neurostimulation methods to target the cerebellum, studies in the 1970–1980s used surgical methods to implant a cerebellar pacemaker in patients with schizophrenia (17–20). This approach was motivated by animal research showing that the deep cerebellar nuclei are connected to the limbic system and play an important role in affective processing (21). As part of this approach, the pacemaker was implanted into the left side of a patient’s chest and connected to electrodes on the cerebellar surface. A battery-operated stimulator worn by the patient then delivered the stimulus through an antenna taped to the skin. During stimulation, electroencephalography (EEG)-based auditory and somatosensory evoked potentials were reduced in amplitude (18). While some participants did benefit, the cerebellar pacemaker was not always well-tolerated by patients. These studies were fraught with high rates of non-compliance (17–19), with many patients refusing to wear the pacemaker and multiple incidents of device and antenna breakage. Long-term use also led to frontal headaches and vertigo in a subset of patients (19). Critically, these invasive surgical procedures were also inherently associated with serious surgical risks, including air embolisms, formation of cerebrospinal fluid fistula, shifting of implanted electrodes, acute inflammation, and/or seizures (18, 20).

### 1.2 Non-invasive approaches to stimulation

**FIGURE 1:**
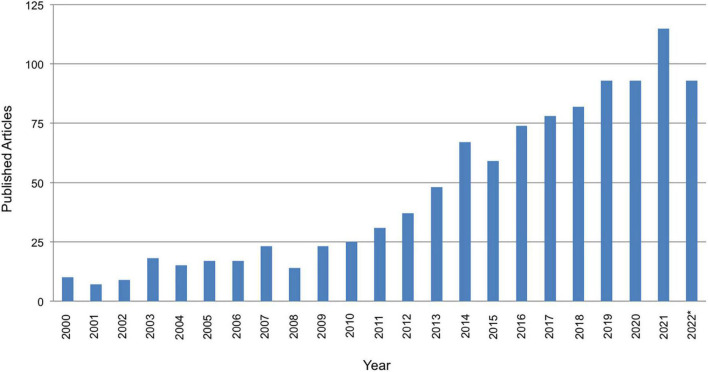
PubMed search of published articles on by year January 1, 2000 to October 11, 2022 for transcranial AND cerebell* AND stimulation. Note that the total number of articles for year 2022 is lower as the year was not over at the time the search was conducted.

Recent technological advances led to more effective and tolerable, non-invasive brain stimulation methods that can be safely applied to the cerebellum (22, 23). Consequently, there has been exponential growth in studies using the methods depicted in Figure 1 such as repetitive transcranial magnetic stimulation (rTMS), transcranial direct current stimulation (tDCS), and transcranial pulsed current stimulation (tPCS). TMS consists of the generation of a brief, high-intensity magnetic field by passing a brief electric current through a magnetic coil (24, 25). This magnetic field will either excite or inhibit a targeted region underneath the coil. Intermittent theta burst stimulation (iTBS) is a newer form of rTMS that provides a two-second train of bursts (30 pulses) every 10 s, and is the most commonly used method of cerebellar stimulation (26). Relative advantages of iTBS vs. traditional rTMS is that stimulation sessions are shorter, utilize a lower threshold intensity, and have more long-term excitatory meta-neuroplastic effects (27, 28). Another form of TBS that has been used is continuous TBS (often referred to as cTBS, though we note that this acronym has also been used to refer to cerebellar TBS in some studies). Continuous TBS provides a burst of 3 pulses at 50 Hz for either 20 or 40 s (29). While iTBS is considered facilitatory, continuous TBS is thought to suppress cortical excitability (29, 30). Although even continuous TBS, which is considered an inhibitory protocol, can lead to downstream increases in functional connectivity between brain areas (31).

In addition to magnetic stimulation approaches, electrical cerebellar stimulation methods have also been used, albeit not as commonly. Through the use of anodal or cathodal stimulation, tDCS can modulate cortical excitability of targeted neural circuits by either increasing or decreasing intrinsic neural firing (32). tDCS entails delivery of a weak direct current through a surface scalp electrode over the cerebellum (33). It is thought that tDCS modulates neural activity in a polarity-specific manner (32, 34). Although, tDCS has poorer focality than TMS (35). tPCS, another method of transcranial electrical stimulation that is used to directly modulate neuro-oscillations, delivers a pulsating current of a specific frequency over a targeted area (36). Advantages of tDCS and tPCS are that these devices are typically inexpensive, battery-operated, and portable (35). Thus, tDCS and tPCS can be used for in-home treatment (32, 37). Initial research across six clinical trials showed the feasibility and tolerability of implementing repeated sessions of at-home tDCS with remote supervision (23). These different approaches highlight the complex and far-reaching capabilities of neurostimulation; capabilities of which we are just beginning to understand.

### 1.3 The cerebellum as a target of brain stimulation

Previous brain stimulation studies in schizophrenia have typically targeted cortical regions, such as the frontal cortex and the motor cortex, in an effort to improve positive symptoms (38, 39), negative symptoms (40–42), and cognitive deficits (42, 43). The rationale for the cerebellum as a brain stimulation target in schizophrenia is at least twofold: first, there is increased knowledge documenting relationships between cerebellum abnormalities and clinical features of schizophrenia (8–10), and second, the cerebellum has several unique attributes that make it an attractive stimulation site, such as its immense and distributed connections throughout the cortex, impressive processing capabilities, and inherent plasticity (33, 44–46). The cerebellum contains over 50–80% of the brain’s neurons while only accounting for 10% of the brain’s volume (47, 48). To accommodate all of these neurons within a small volume, the large number of cells is packed in a columnar array with modules that are perpendicular to the cortical surface and parallel to each other (3–5). This organizational structure is conducive to massive parallel processing (3–5) and has been likened to a biological equivalent of a modern microprocessor chip (5). The cerebellum is also located immediately below the skull making it a convenient site for electrode placement (44). Moreover, the cerebellar cortex has been found to be highly responsive to electrical and magnetic stimulation (44).

One of the critical advantages of cerebellar stimulation lies in the potential for modulating cerebello-cerebral circuits, and in turn, impacting cognitive and behavioral functions that depend on these distributed circuits (12, 14). The cerebellum is structurally and functionally connected to numerous cortical and subcortical regions (6, 49–51), with closed parallel loops that link the cerebellum to distant cortical regions (6, 12, 52). Consequently, the cerebellum has been described “as a window to the whole brain” (15). By stimulating the cerebellum, researchers can indirectly modulate dysfunctional cortical circuitry *via* cerebello-cerebral circuits (14, 15, 44). Additionally, it has been posited that cerebellar stimulation could lead to long-lasting modulatory effects in schizophrenia through the induction of cerebellar plasticity (53). It is thought that the cerebellum has both long-term synaptic and non-synaptic plasticity (45, 46, 54), both of which drive new learning (46, 54). This notion is supported by evidence of induced plasticity in cerebellum-involved pathways (e.g., cerebello-premotor-motor and cerebello-frontal pathways) following rTMS (55, 56).

### 1.4 Purported mechanisms of cerebellar stimulation

The precise mechanisms of non-invasive stimulation of the human cerebellum are unknown. One theory is that at least some forms of neurostimulation, like TMS and tDCS, modulate the excitability of Purkinje cells (PCs), a class of GABAergic inhibitory neurons found in the superior cerebellum (53). PCs are large cerebellar output neurons that play a central role in the cerebellar cortical circuit by modulating activity in the deep cerebellar nuclei outflow. Pre-clinical findings have shown that rTMS using a low-intensity current in mice can alter dendritic and spine morphology of Purkinje cells (25). Similarly, it was recently shown that while tDCS-induced electrical field changes can reach deep cerebellar nuclei, PCs were the most sensitive cell type to tDCS (57). More specifically, tDCS anodal stimulation has an excitatory effect that increases output of PCs, and consequently, leads to greater inhibition of cerebello-cerebral pathways; cathodal stimulation has the opposite effect, and is thought to be inhibitory to PCs, leading to disinhibition of the cerebral cortex (14, 58).

As noted above, cerebellar neurostimulation has the potential to induce cerebellar plasticity as seen in healthy individuals (55) and in stroke patients (59). TMS has been found to effect such change through the induction of cerebellar long-term plasticity (LTP) (60), and it is thought that tDCS effects change *via* a comparable system (15). The most common form of LTP, and its inverse long-term depression (LTD), depend on activation of *N*-methyl-D-aspartate receptors (NMDAR) (28, 61). The relationship between LTP and NMDAR is evident by the fact that plasticity-inducing effects of neurostimulation effects can be blocked by the administration of NMDAR antagonists, like memantine and dextromethorphan (27, 62). Both LTP and NMDAR abnormalities are also implicated in the pathophysiology of schizophrenia (63–68).

Additionally, the effects of neurostimulation can extend throughout the brain, beyond the initial stimulation target. For instance, tDCS effects may influence activity in both the specific target region and multiple network systems by way of increasing/decreasing release of monoamine neurotransmitters, like dopamine, onto circuits that do not even involve the anodal stimulation site (69, 70). Studies of rTMS (including iTBS) also show downstream effects of stimulation on broader networks (64). These downstream effects on cerebello-cerebral networks are thought to be beneficial in ameliorating clinical symptoms and cognitive deficits.

### 1.5 Implications of cerebellar stimulation in schizophrenia

Over two decades ago, Andreasen et al. (71) called attention to the cerebellum through their cognitive dysmetria hypothesis, which posits a deficit in the underlying neural system responsible for coordinating the processing, prioritization, and expression of information among people with schizophrenia (71). Since then, a number of other mechanistic hypotheses involving the cerebellum have been proposed to explain clinical phenomena in psychosis (72). These studies have not only led to new discoveries regarding the cerebellum’s role in the pathophysiology of schizophrenia, but also have implications for treating schizophrenia.

An initial systematic review reported on 10 studies (3 randomized controlled trials [RCTs], 3 open-label studies, and 2 case reports) of cerebellar stimulation in schizophrenia (26). These studies found that cerebellum stimulation produced clinical changes in negative and depressive symptoms, as well as cognitive functioning domains. Critically, cerebellum modulation showed potential for alleviating schizophrenia symptoms that are less responsive to antipsychotic medications, i.e., negative symptoms (73). These promising findings garnered further enthusiasm for cerebellum stimulation as a treatment for schizophrenia (11, 13), as evidenced by multiple published studies following Escelsior’s initial review and several ongoing clinical trials.

### 1.6 Aims

This manuscript provides a systematic update regarding the effects of cerebellar stimulation in schizophrenia. We discuss the effects on clinical symptoms, cognition and behavior, functional brain networks and underlying neuro-oscillations, movement, and physiology. We also review the tolerability of this intervention method for individuals with schizophrenia. We close by discussing issues and technical considerations regarding implementation of cerebellar stimulation as well as recommendations and future directions.

## 2 Materials and methods

### 2.1 Abstract and article search

This systematic review was pre-registered on PROSPERO (CRD42022346667) and adheres to PRISMA guidelines (74). JPYH searched research databases (PubMed, Scopus, and PsycInfo) to identify published articles from inception until October 11, 2022. All empirical studies (i.e., excluding reviews and meta-analyses) that reported on the effects of cerebellar stimulation, obtained by physical or pharmacological means (e.g., electric or magnetic stimulation or *in situ* injection), among animal models of schizophrenia, patients with schizophrenia-spectrum disorders, and individuals at risk of psychosis were included in the review. Articles were included if they included just an active stimulation arm comparing baseline to post-stimulation, or if they included a comparison of active cerebellar stimulation to a sham or active control condition. Unpublished papers and clinical trials were excluded from the systematic review. We systematically searched titles and abstracts using the following Boolean search terms: schizoph* AND cerebell* AND (modulation OR intervention OR stimulation OR transcranial OR TMS OR tDCS OR TBS OR tACS OR injection). References from all included papers as well as a previous systematic review (26) were also evaluated. This screening process was followed by independent full-text screening of all potentially relevant articles and data extraction by JPYH and SVA. Extracted study data included author name and year, description and size of study sample, type of research design (i.e., RCT, open-label uncontrolled study, pilot study, case report, or preclinical study), names and types of measures, assessment timepoints, cerebellar stimulation and sham parameters, and study outcomes (i.e., clinical, cognitive, behavioral, connectivity and oscillatory, movement, physiological, and tolerability/side effects).

### 2.2 Risk of bias and quality assessment

Quality of included studies was classified based on Nathan and Gorman’s criteria (75) for rating the methodological rigor of study designs. According to this classification system, there are six levels of studies from Type 1 (most rigorous) to Type 6 (least rigorous). Type 1 studies are double-blind, randomized, prospective, controlled clinical trials. These studies involve comparison of randomized groups, state-of-the-art diagnostic and assessment methods, appropriate analytic methods, clear exclusion and inclusion criteria, and adequate sample size. Type 2 studies are clinical trials that lack some of the rigorous criteria of a Type 1 study, such as small sample sizes, lack of clearly defined inclusion and exclusion criteria, and problems with the randomization protocol. Type 3 studies are open treatment studies and include designs such as pilot and case-control studies. These studies are often methodologically limited by observer bias, retrospective recall error, and uncontrolled data collection. Type 4 studies entail sophisticated analysis of secondary data analyses (e.g., meta-analysis). Type 5 studies are review studies that do not include data analysis. Type 6 studies are case studies, opinion pieces, and essays. Based on the article inclusion criteria for the current study, Type 4 and 5 studies were not included.

## 3 Results

### 3.1 Study selection

**FIGURE 2:**
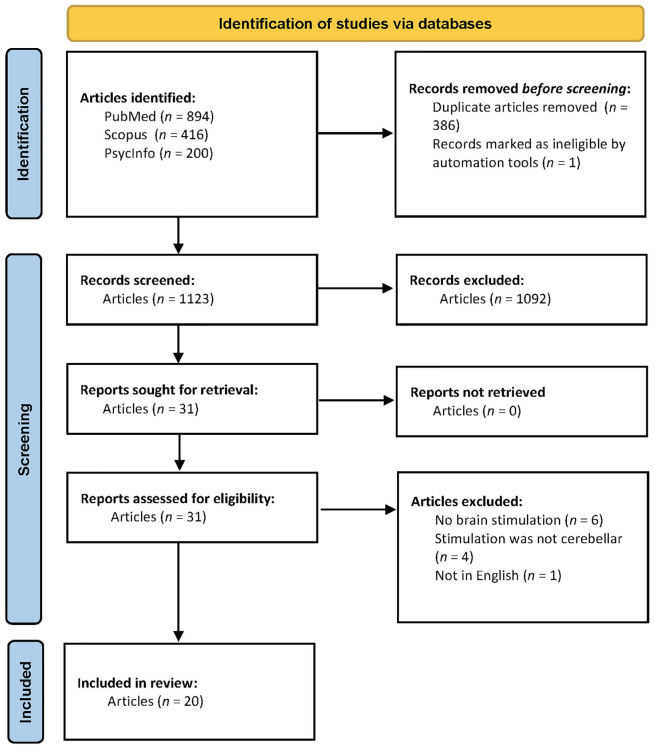
PRISMA flow diagram regarding study inclusion and exclusion.

The systematic literature search yielded a total of 1,510 published studies (see Figure 2 for PRISMA diagram). Of these, JPYH identified 31 published articles for full review. No additional articles were identified from review of study bibliographies. Based on full-article review, JPYH and SVA independently identified 20 published articles for this review (see Tables 1, 2).

**TABLE 1:** Characteristics of clinical studies reporting on cerebellar stimulation in schizophrenia.

References	Participants	Study design	Brain stimulation	Results^a^	Study level
Boechat-Barros et al. (84)*	Four chronic SZ with tardive dyskinesia	**Design:** Pilot study **Measures:** PANSS, CGI-SCH (clinical); AIMS (physiological) **Time points:** baseline (T0), after the 1st session (T1), after the 5th session (T2), post-stimulation 3 months (T3)	**Active:** Five tDCS sessions on consecutive days targeting the central cerebellum at 2 cm below the inion (2 milliamps for 20 min)	**Clinical:** Across time, SZ patients showed numerical decreases in AIMS clinician-rated tardive dyskinesia (T1 minus T0: −22.9%; T2 minus T0: −33.5%) and CGI−SCH global symptoms (T1 minus T0: −19.9%; T2 minus T0: −32.8%). SZ patients also showed decreases in PANSS positive (T2 minus T0: −22.9%), negative (T2 minus T0: −27.8%), general (T2 minus T0: −36.5%), and total (T2 minus T0: −32.5%) symptoms. Two patients showed increases in CGI-SCH global and PANSS symptoms at T3. **Physiological:** Reductions in tardive dyskinesia remained at T3. **Tolerability/Side Effects:** Mild side effect reported (i.e., skin burn under the cathode electrode).	6
Basavaraju et al. (90)*	60 SZ with at least moderate negative symptoms, i.e., ≥3 on each SANS global item [2 participants were previously reported on in Basavaraju et al. (91)]	**Design:** Randomized clinical trial; 30 SZ active and 30 SZ sham **Measures:** SANS, SAPS, CDSS (clinical); MATRICS (cognitive); rsfMRI (connectivity); ataxia (movement); pulse rate, blood pressure (physiological) **Time points:** baseline (T0), 6 days (T1), 6 weeks (T2)	**Active:** 10 rTMS-iTBS sessions targeting cerebellar vermis identified through neuronavigation (2 sessions daily spaced 4 h apart for 5 days; 20 trains of 2 s on and 8 s off cycle containing 3-pulse 50 Hz bursts at theta frequency every 200 ms; total of 6,000 pulses; figure-of-eight coil) **Sham:** 10 sessions that produced a sound comparable to rTMS-iTBS but without magnetic stimulation (2 sessions daily spaced 4 h apart for 5 days)	**Clinical:** No specific effect of active stimulation for SANS negative, SAPS positive, or CDSS depressive symptoms; rather, both groups improved on all symptoms over time. **Cognitive:** No significant effect of active stimulation vs. sham. Both groups improved on multiple cognitive measures over time. **Connectivity:** Following active stimulation only, resting-state functional connectivity increased between the cerebellum and right inferior frontal gyrus, right pallidum, and right frontal pole. **Movement:** No specific effect of active stimulation. Both groups had decreased extrapyramidal symptoms and ataxia over time. **Physiological:** No specific effect of active stimulation. Both groups had decreased diastolic blood pressure over time. **Tolerability/Side effects:** Two participants in the active arm reported mania/hypomania symptoms (also in the 2020 paper). One additional participant in the active arm reported neck muscle contraction and ensuing tolerable neck pain during stimulation.	1
Chauhan et al. (82)*	30 treatment-resistant SZ	**Design:** Randomized placebo-controlled trial; 16 SZ active and 14 SZ sham **Measures:** PANSS, BPRS, CGI (clinical); SCoRS (cognitive); SAS (movement) **Time points:** baseline (T0), after session 10 (T1), post-stimulation 2 weeks (T2)	**Active:** 10 rTMS-iTBS sessions targeting the cerebellar vermis and positioned using the 10–20 EEG system (2 sessions daily spaced ≥ 30 min apart for 5 days; 20 trains of 10 bursts given at 8 s intervals containing 3-pulse 50 Hz bursts at 5 Hz; total of 6,000 pulses; figure-of-eight coil) **Sham:** 10 sessions carried out by an active/sham coil that had both sound and scalp contact similar to active stimulation (2 sessions daily spaced ≥ 30 min apart for 5 days)	**Clinical:** No specific effect of active stimulation vs. sham on symptom severity. Both groups had decreased psychiatric symptoms as indicated by PANSS, BPRS, and CGI scores at T1 and/or T2. **Cognitive:** No specific effect of active stimulation vs. sham. Both groups improved on SCoRS cognition over time. **Movement:** No effect of active stimulation vs. sham. No change in SAS symptoms over time. **Tolerability/Side effects:** Five patients in the active arm and two in the sham arm reported headaches during the first few sessions that were alleviated with analgesics.	2
Zhu et al. (83)*	64 SZ	**Design:** Multicenter, randomized, sham-controlled, double-blind clinical trial; 32 SZ active and 32 SZ sham **Measures:** PANSS **Time points:** baseline, end of treatment, and post-stimulation 2, 6, 12, and 24 weeks	**Active:** 10 rTMS-iTBS sessions targeting the cerebellar vermis at 1 cm below the inion (5 days a week for 2 weeks; 20 trains of 10 bursts given at 8 s intervals containing 3-pulse 50 Hz bursts at 5 Hz; total of 6,000 pulses; figure-of-eight coil) **Sham:** 10 sessions with coil flipped 180 or 90° using the same pulse sequence to realize the effect of sham stimulation (5 days a week for 2 weeks)	**Clinical:** Negative symptom scores decreased at each time point in the active group only (baseline vs. post stimulation *d* = −0.27; baseline vs. 24-week follow-up *d* = −0.67). PANSS total, positive, and general psychotic symptoms also decreased over time with the lowest scores at 24 weeks. **Tolerability/Side effects:** Three patients in the active arm reported mild dizziness, pain, nausea, and other symptoms after the first session. These symptoms were relieved after a short break, and there were no other side effects in subsequent sessions.	1
Basavaraju et al. (91)*	Two SZ with at least moderate negative symptoms (i.e.,≥3 on the SANS global ratings)	**Design:** Case study **Measures:** SANS, SAPS, YMRS **Time points:** baseline (T0), 6 days (T1), 6 weeks (T2)	**Active:** 10 rTMS-iTBS sessions targeting the cerebellar vermis identified through neuronavigation (2 sessions daily spaced 4 h apart for 5 days; 20 trains of 2 s on and 8 s off cycle containing 3-pulse 50 Hz bursts at theta frequency every 200 ms; total of 6,000 pulses; figure-of-eight coil)	**Clinical:** Across time, SZ patients showed numerical decreases in negative (T1 minus T0: −13.0; T2 minus T0: −30.5) and positive (T1 minus T0: −3.5; T2 minus T0: −4.5) symptoms and increases in manic symptoms (T1 minus T0: 4.5; T2 minus T0: 18). **Tolerability/Side effects:** Two participants in the active arm showed symptoms of mania/hypomania.	6
Laidi et al. (85)*	One SZ	**Design:** Case study **Measures:** PANSS, AHRS (clinical); free and cued recall, verbal episode memory tests, WAIS digit span, WAIS spatial span, Stroop test, D2 test of attention (cognitive); eye blink conditioning (behavioral) **Time points:** pre-stimulation and post-stimulation	**Active:** 10 tDCS sessions on consecutive days targeting the posterior cerebellum (2 sessions daily spaced 1 h apart for 5 days; 2 mA for 25 min)	**Clinical:** There was no change in PANSS and AHRS psychotic symptoms following treatment. **Cognitive:** After treatment, the patient showed broad improvements in cognitive functions, i.e., verbal episodic memory, short term memory, working memory, executive functioning, and attention. **Behavioral:** After treatment, the patient showed clear improvement of eye blink conditioning. Before treatment, the patient could not be conditioned over the eye blink conditioning session, and after cerebellar tDCS, the patient showed progressive conditioning from block to block. **Tolerability/Side effects:** No significant side effects reported.	6
Brady et al. (89)	11 SZ	**Design:** Double-blind, randomized sham-controlled trial; 8 active and 3 sham **Measures:** PANSS (clinical); rsfMRI (connectivity) **Time points:** pre-stimulation and post-stimulation	**Active:** 10 rTMS-iTBS sessions targeting the cerebellar vermis identified using the Brainsight frameless stereotaxic system (2 sessions daily spaced 4 h apart for 5 days; 10 bursts given at 10 s intervals containing 3-pulse 50 Hz bursts at 5 Hz; total of 6,000 pulses; figure-of-eight coil) **Sham:** sham rTMS-iTBS sessions targeting the cerebellar vermis identified using the Brainsight frameless stereotaxic system (2 sessions daily spaced 4 h apart for 5 days)	**Clinical:** Reduced PANSS negative symptom severity after stimulation vs. sham (*d* = −0.91). **Connectivity:** Increased cerebellar-dorsolateral prefrontal cortex connectivity after stimulation (*d* = 0.25). **Correlation:** Increased cerebellar-dorsolateral prefrontal cortex connectivity correlated with PANSS negative symptom reductions (*r* = 0.81).	2
Singh et al. (93)	Nine SZ	**Design:** Double-blind, randomized, sham-controlled trial	**Active:** One tPCS session targeting the cerebellar vermis at 1 cm below	**Oscillatory:** Theta oscillations were significantly larger following theta frequency	2
		**Measures:** EEG (oscillatory); interval timing task (behavioral); Montreal Cognitive Assessment, Trail Making Task, verbal fluency, and digit span (cognition) **Time points:** pre-stimulation and post-stimulation	the inion at theta frequency (20 min at 1 mA) **Active Control:** One tPCS session targeting the cerebellar vermis at 1 cm below the inion at delta frequency (20 min at 1 mA)	cerebellar tPCS, but not delta tPCS, in the midfrontal region. **Behavioral:** Neither theta nor delta tPCS was associated with changes in the interval timing task. **Cognition:** There were no significant changes for cognitive tasks after tPCS.	
Gupta et al. (94)	24 non-clinical psychosis (i.e., high schizotypy) scoring in the top 15th percentile on the CAPE and 18 UCS scoring in the bottom 15th percentile on the CAPE	**Design:** Randomized, double-blind, sham-controlled crossover trial **Measures:** pursuit rotor task (cognition) **Time points:** baseline stimulation and 1-week stimulation	**Active:** One tDCS session targeting the cerebellar midline at 1–2 cm below the inion (25 min at 2 mA) **Sham:** One sham session targeting the cerebellar midline at 1–2 cm below the inion (30 s at 2 milliamps)	**Cognition:** Non-clinical psychosis showed a greater rate of learning in the active condition vs. sham compared to the control group (η^2^ = 0.10). In the active condition, the non-clinical psychosis group performed the task at a level that was comparable to the UCS group, with no difference between groups in the active condition.	1
Garg et al. (81)	40 SZ	**Design:** Randomized rater blind-sham controlled study; 20 active and 20 sham **Measures:** PANSS, CDSS (clinical) **Time points:** pre-treatment, after 10th session, post-stimulation 2 weeks	**Active:** 10 rTMS (theta range) sessions over 2 weeks targeting the cerebellar vermis at 1cm below the inion (20 pulses each for 30 trains, 10 trains each of 5, 6, and 7 Hz followed each other sequentially; train duration for 5 Hz stimulation was 4 s, for 6 Hz was 3.33 s, and for 7 Hz was 2.857 s and the inter-train interval was kept constant at 20 s; total 6,000 pulses; figure-of-eight coil) **Sham:** 10 sham sessions over 2 weeks (sound and scalp contact were roughly similar to active stimulation)	**Clinical:** There was an effect of active vs. sham indicated by reductions in PANSS total symptoms, PANSS negative symptoms, and CDSS depressive symptoms. Yet, when baseline scores were included as covariates, the significant treatment effect on PANSS and depressive symptoms were no longer significant. The time effect for PANSS positive and general symptoms was significant. **Tolerability/Side effects:** No major side effects were reported. Five patients reported headaches that responded to analgesics. One patient reported excessive sleepiness after each session.	1
Tikka et al. (86)	11 recent-onset SZ	**Design:** Open-label uncontrolled study **Measures:** PANSS, CDSS (clinical); EEG (oscillatory) **Time points:** baseline and post-stimulation	**Active:** 10 rTMS sessions (theta range) targeting the cerebellar vermis at 1 cm below the inion and positioned using the 10–20 EEG system (5 days per week for 2 weeks; 30 pulses each for 20 train at frequencies of 5, 6, and 7Hz; total of 6,000 pulses; angled double-cone coil)	**Clinical:** Reduction in PANSS negative (Wilcoxon ES = 0.66) and total symptoms (Wilcoxon ES = 0.65), as well as CDSS depression symptoms (Wilcoxon ES = 0.75) following stimulation. There were no significant changes for PANSS positive symptoms or general psychopathology **Oscillatory:** Reduction of gamma spectral power in left temporal (Wilcoxon ES = 0.83) and left frontal (Wilcoxon ES = 0.73), though the latter did not survive multiple comparison correction. **Correlation:** Percent reduction in PANSS negative symptoms correlated with percent reduction in left temporal (rho = 0.74) and left frontal gamma power (rho = 0.78). Percent reduction in CDSS depressive symptoms correlated with percent reduction in left frontal gamma power (rho = 0.85).	3
Garg et al. (92)	One treatment-resistant SZ	**Design:** Case study **Measures:** PSYRATS-AH, PANSS hallucination score (clinical) **Time points:** baseline, day 5, post-stimulation 2 and 8 weeks	**Active:** Four rTMS sessions over 5 days targeting the cerebellar vermis at 1 cm below the inion and positioned using the 10–20 EEG system (20 trains of 30 pulses at 5 Hz for the first 7 trains, 6 Hz for the next 7 trains, and 7Hz for the final 6 trains; total of 2,400 pulses; figure-of-eight coil)	**Clinical:** Worse auditory hallucination frequency and hallucination-associated distress. Numerical increase in PSYRATS-AH and PANSS hallucination scores at termination of treatment. Elevated scores remained after 2 weeks, and returned to baseline at 8 weeks. **Tolerability/side effects:** Discontinued treatment after 4 sessions (instead of 10) due to symptom exacerbation.	6
Garg et al. (88)	One first-episode SZ	**Design:** Case study **Measures:** PANSS (clinical); EEG (oscillatory) **Time point**s: baseline (T0), post-stimulation 2 (T1), 4 (T2), and 6 (T3) weeks	**Active:** 10 rTMS (theta range) sessions over 2 weeks targeting the cerebellar vermis at 1 cm below the inion and positioned using the 10–20 EEG system (20 trains of 30 pulses at 5 Hz for the first 7 trains, 6 Hz for the next 7 trains, and 7 Hz for the final 6 trains; total of 6,000 pulses; figure-of-eight coil)	**Clinical:** Decreased PANSS total (T1 minus T0: −38), PANSS anergia (T1 minus T0: −11), and PANSS thought disorder (T1 minus T0: −10) scores. Score decreases maintained at 4 and 6 weeks post-stimulation. **Oscillatory:** Post rTMS EEG showed significant increases in gamma spectral power in the left frontal, right frontal, and left occipital regions, as well as significant decreases in gamma spectral power in the left temporal region. **Tolerability/side effects:** No side effects reported.	6
Demirtas-Tatlidede et al. (87)	Eight treatment-resistant SZ with moderate-to-severe illness severity	**Design:** Open-label uncontrolled study **Measures:** PANSS, CGI, CDSS, POMS, VAS (clinical); attention, working memory, long-term memory, speed of processing, executive functions, visuospatial skills, and motor functioning (cognitive); diastolic and systolic blood pressure, and heart rate/pulse (physiological) **Time points:** baseline, post-stimulation, and post-stimulation 1 week	**Active:** 10 rTMS-iTBS sessions targeting the cerebellar vermis identified using the Brainsight frameless stereotaxic system (2 sessions daily for 5 days; 20 trains of 10 bursts given at 8 s intervals containing 3-pulse 50 Hz bursts at 5 Hz; total of 6,000 pulses; figure-of-eight coil)	**Clinical:** Patients showed a decrease of PANSS negative symptoms following stimulation; *post hoc* comparisons showed differences between baseline vs. post-stimulation (*d* = 0.69) and baseline vs. 1-week follow-up (*d* = 0.60). No effect of stimulation on PANSS total, positive or general psychotic symptoms or CGI global impression. Patients showed an increase in CDSS depressive symptoms following stimulation. *Post hoc* comparisons showed differences between baseline vs. post-stimulation (*d* = 0.72). POMS showed a similar pattern in results, but did not reach significance. Happiness showed an increase, with differences between baseline vs. post-stimulation (*d* = 1.39) and baseline vs. 1-week follow-up (*d* = 1.20). Sadness showed a decrease, with differences between baseline vs. post-stimulation (*d* = 1.15). Alertness showed an increase, with differences between baseline vs. 1-week follow up (*d* = 0.80). Other mood ratings showed no significant effects. **Cognitive:** After stimulation, patients had improved performance on the continuous performance test, evidenced by fewer omissions during memory (*d* = 0.78) and interference conditions (*d* = 1.04), when for performance at baseline vs. 1-week follow-up. Spatial span forward performance showed an increase between baseline vs. post-stimulation and 1-week follow-up (*d* = 0.69). Further, patients improved in their organization of the Rey–Osterrieth Complex figure at delay for between baseline vs. 1-week follow-up (*d* = 0.68). There was no decrease in performance on any cognitive domain after cerebellar brain stimulation. **Physiological:** There were no serious cardiovascular events. Diastolic blood pressure increased immediately post-stimulation and five minutes after, but soon returned to baseline levels. No significant change for systolic blood pressure or pulse.	3
				**Tolerability/side effects:** Side effects were mild and included neck pain and headache which both responded to analgesics, discomfort at stimulation site, and light-headedness. Patients reported no new symptoms or worsening of existing symptoms.	
Daskalakis et al. (100)*	10 SZ; 10 UCS	**Design:** Open-label uncontrolled study **Measures:** Electromyography **Time points:** post-stimulation	**Active:** TMS to the center of the right cerebellar hemisphere; figure-of-eight coil (conditioning stimulus)	**Electromyography:** SZ showed deficits in cerebellar inhibition compared with UCS (*d* = 1.02).	3
Heath et al. (19)*	15 treatment-resistant SZ; 5 patients appropriate for this study with psychotic behavior after organic brain syndrome [5 of these participants were previously reported on in Heath (17), and all 20 were previously reported on in Heath et al. (18)]	**Design:** Open-label uncontrolled study **Measures:** Clinical summaries **Time points:** longitudinal follow-up up to 54 months	**Active:** A pacemaker was implanted into the left side of a patient’s chest and connected to electrodes stimulating the superior surface to the inferior surface of the cerebellar vermis. A battery-operated stimulator worn by the patient delivered an electrical stimulus through an antenna taped to the skin.	**Clinical:** Among treatment-resistant SZ, 3 had significant functional improvement (living at home, no medications, no psychotic symptoms), 3 had moderate improvement and were functioning outside of the hospital (low medication dosage), 2 had minimal improvement, and 7 showed no improvement (6 of these 7 refused to wear the stimulator). Among patients with psychotic behavior after organic brain syndrome, 2 had significant improvement, 1 had moderate improvement, 1 had minimal improvement, and 1 showed no improvement. **Tolerability/side effects:** Six patients refused to wear the stimulator. There were also issues with hardware being defective in many patients.	6
Correa et al. (20)*	12 SZ who were determined to have disabling emotional symptoms; 1 patient with psychotic behavior after organic brain syndrome	**Design:** Open-label uncontrolled study **Measures:** Clinical summaries **Time points:** longitudinal follow-up varied by patient	**Active:** A pacemaker was implanted into the left side of a patient’s chest and connected to electrodes stimulating the vermis and paravermis regions.	**Clinical:** At follow-up, 1 SZ was rated as excellent (clearing of hallucinations/delusions, improvement of blunted affect and disorganized thinking), 4 were rated as good (decrease in psychotic symptoms), 1 was rated as fair (no change in hallucinations/delusions, but improvement in affect and disorganized thinking), 4 were rated as poor (no change in symptoms; 3 of the 4 showed long-term improvement), and 2 were lost to follow-up. The patient with psychosis after organic brain syndrome showed some improvement in their emotions. **Tolerability/side effects:** There were some surgical complications including air embolisms, formation of cerebrospinal fluid fistula, shifting of implanted electrodes, and headaches.	6
Heath et al. (18)	15 treatment-resistant SZ; 5 patients with psychotic behavior after organic brain syndrome [5 of these participants were previously reported on in Heath (17)]	**Design:** Open-label uncontrolled study **Measures:** Clinical summaries **Time points:** longitudinal follow-up between 3 and 27 months	**Active:** A pacemaker was implanted into the left side of a patient’s chest and connected to electrodes stimulating the superior surface to the inferior surface of the vermis. A battery-operated stimulator worn by the patient then delivers an electrical stimulus through an antenna taped to the skin.	**Clinical:** Among treatment-resistant SZ patients, 2 had significant improvement (living at home, no medications, no psychotic symptoms), 6 had moderate improvement and were functioning outside of the hospital (low medication dosage), 3 had minimal improvement, and 4 showed no improvement (3 of these 4 refused to wear the stimulator). Among patients with psychotic behavior after organic brain syndrome, 4 had significant improvement and 1 showed no improvement. In the most effective protocols, electrodes were placed on the surface of the cerebellar vermis. **Tolerability/side effects:** Three patients refused to wear the simulator. There were also issues with antenna breakage and formation of cerebrospinal fluid fistula.	6
Heath et al. (17)*	Five treatment-resistant SZ who had been pronounced incurable by ≥2 physicians	**Design:** Open-label uncontrolled study **Measures:** Clinical summaries **Time points:** longitudinal follow-up between 3 and 16 months	**Active:** A pacemaker was implanted into the left side of a patient’s chest and connected to electrodes on the cerebellar surface, namely rostral vermal and para vermal regions. A battery-operated stimulator worn by the patient then delivers an electrical stimulus through an antenna taped to the skin.	**Clinical:** Four of five patients showed a significant decrease in psychotic symptoms and in need for neuroleptic medication as well as improvement in functioning. 1 patient, who had a lesion over the stimulation site, showed no improvement and repeatedly destroyed the pacemaker and antenna. **Tolerability/side effects:** One patient refused to wear the stimulator, and repeatedly destroyed the equipment.	6

AHRS, auditory hallucination rating scale; AIMS, abnormal involuntary movement scale; BPRS, brief psychiatric rating scale; CDSS, calgary depression rating scale; CGI-SCH, clinical global impression—schizophrenia; EEG, electroencephalography; iTBS, intermittent theta burst stimulation; MATRICS, measurement and treatment research to improve cognition in schizophrenia cognitive consensus battery; PANSS, positive and negative syndrome scale; POMS, profile of mood states; PSYRATS, psychotic symptom rating scale-auditory hallucination subscale; rsfMRI, resting-state functional magnetic resonance imaging; rTMS, repetitive transcranial magnetic stimulation; SANS, scale for the assessment of negative symptoms; SAPS, scale for the assessment of positive symptoms; SAS, Simpson–Angus extrapyramidal side effects scale; SCoRS, schizophrenia cognition rating scale; SZ, schizophrenia; TBS, theta burst stimulation; tDCS, transcranial direct current stimulation; UCS, unaffected comparison subject; VAS, visual analogue scales (dimensions of mood: happiness, sadness, calmness, anxiety, wellbeing, anger, self-confidence, fear, alertness, and energy); tPCS, transcranial pulsed current stimulation; WAIS, Wechsler adult intelligence scale; YMRS, young mania rating scale.

^a^Effect sizes were included or computed when possible.

*Not included in previous review.

**TABLE 2:** Characteristics of pre-clinical studies reporting on cerebellar stimulation in rat models of schizophrenia.

References	Participants	Study design	Brain stimulation	Results
Parker et al. (95)	Nine rats with dopamine receptor blockade in medial prefrontal cortex	**Design:** Experimental open-label study **Measures:** interval timing task, lever pressing, liquid rewards, or open-field activity (behavioral) **Time points:** post-stimulation	**Active:** Optogenetic stimulation (delta frequency) targeting right lateral cerebellar nuclei projections to the thalamus	**Behavioral:** Optogenetic stimulation of lateral cerebellar nuclei projections at 2 Hz, but not 4, 10, or 20 Hz, rescued behavioral deficits on the interval timing task. There was no clear effect of optogenetic stimulation on lever pressing, rewards or open-field.

### 3.2 Study characteristics

#### 3.2.1 Patient characteristics

Collapsed across included articles, this review included 283 patients with schizophrenia, 24 individuals scoring high on schizotypy scales, 9 rodents whose brains were manipulated to simulate schizophrenia-like deficits, and 28 healthy controls. Of the chronic schizophrenia studies, 3 studies recruited patients with moderate symptoms and 7 studies recruited treatment-resistant patients, as defined as patients whose symptoms were unsuccessfully treated through multiple courses of different antipsychotic medications.

#### 3.2.2 Methodological characteristics

Researchers used a variety of study designs, with 7 studies being RCTs, 8 being open-label uncontrolled studies (1 pilot study), 4 case reports, and 1 preclinical study. Of the non-invasive brain stimulation studies, 9 were longitudinal, with the longest follow-up timepoint at 6 months (but the majority were under 6 weeks). Of all studies involving humans, 4 met criteria for Type 1 (RCTs with a sample ≥ 40), 3 for Type 2 (RCTs with a sample < 40), 3 for Type 3 (open-label uncontrolled studies), and 9 for Type 6 (descriptive and case studies). Note that study level criteria were not applied to the preclinical study because the criteria were based on human standards. Although the cerebellar pacemaker papers were technically open-label uncontrolled studies, they were categorized as Type 6 because of the use of clinical summaries as well as broad and non-specific treatment outcome categories. As can be seen in Table 1, there has been a shift in recent years to include more rigorous research designs, such as RCTs with larger sample sizes. In fact, an additional 9 clinical trials are currently recruiting and have not yet posted results.

Most of the included studies used TMS (11 studies), with 6 studies using iTBS, specifically. Three studies used tDCS, 1 used tPCS, and 4 used cerebellar pacemakers (all prior to 1982). The preclinical study used delta-frequency optogenetic stimulation targeting the right lateral cerebellar nuclei. Of the non-invasive brain stimulation studies, 12 studies used repetitive magnetic pulses, and 10 studies included multi-session designs with 10-session designs most commonly used. Eleven studies stimulated the cerebellar midline/vermis.

### 3.3 Effects of cerebellar stimulation on clinical symptoms and mood

Cerebellar pacemaker studies were the pioneer studies that tested the effectiveness of cerebellar stimulation on alleviating symptoms and improving functioning in schizophrenia. These treatment-resistant, small sample studies showed modest improvements in clinical symptoms and functioning (e.g., living at home, little to no medication, little to no psychotic symptoms) at different follow-up periods (17–20); however, results are difficult to interpret since assessments were not standardized and there were challenges arising from faulty equipment and low treatment tolerability. This approach is rather controversial and no longer recommended (76). Nonetheless, cerebellar pacemaker studies set the stage for the current non-invasive stimulation methods. Due to the increased scientific rigor of more modern studies, we weigh these studies more heavily in our results summary.

Of the studies utilizing non-invasive brain stimulation, 12 examined the effects of cerebellar stimulation on clinical symptoms. The most consistently examined clinical domain was psychotic symptoms, with all 12 studies including measures of psychotic symptoms; 10 studies specifically used the total and/or the positive, negative, and general psychopathology subscales from the Positive and Negative Syndrome Scale (PANSS) (77). Three other studies looked at psychotic symptoms using the Scale for the Assessment of Negative Symptoms (SANS) (78) and Scale for the Assessment of Positive Symptoms (SAPS) (79). Additionally, three studies used a measure of overall clinical impression and four used depression and mood inventories, including the Calgary Depression Scale for Schizophrenia (80) and Visual Analogue Scales.

#### 3.3.1 Total symptoms

Seven studies examined the impact of cerebellar stimulation on total symptom scores, which are a combination of negative, positive, and general pscyhopathology symptoms. One study found a specific effect of active rTMS in reducing total symptoms, but this effect was not significant when accounting for baseline total symptom level (81). Several rTMS and iTBS studies observed reductions in total symptoms for participants in both the active and sham arms (81–83). Similarly, several studies with only an active rTMS or iTBS stimulation arm found reductions in total post-stimulation symptoms (84–86) but see Demirtas-Tatlidede et al. (87). These non-specific treatment effects were maintained at 3 months follow-up for five people with schizophrenia who took part in a case study using rTMS or a pilot study using tDCS (84, 88).

#### 3.3.2 Negative symptoms

Nine studies examined negative symptoms associated with psychosis. Multiple RCTs (*N*s ranging 11–64) with active/sham rTMS stimulation protocols (some implementing iTBS, specifically) observed significant Stimulation X Time interactions for PANSS negative symptoms (81, 83, 89). More specifically, participants who received active iTBS had significant negative symptoms reductions compared to those who received sham. Although other iTBS studies found significant improvements in negative symptoms for both the active and sham conditions (82, 90). Studies that included only an active stimulation arm reported decreases in negative symptoms (tDCS, rtMS, or iTBS) (84, 86–88), with evidence that these effects were maintained for as long as 24 weeks (84, 88).

#### 3.3.3 Positive symptoms

Nine studies examined the impact on positive symptoms. Three studies observed non-specific iTBS effects on positive symptoms, with significant reductions for participants in the active and control study arms (82, 83, 90). Studies with only an active stimulation arm (rTMS, iTBS, or tDCS) reported mixed results, with some finding no change (86, 87), others finding a reduction (84, 88, 91), and one case study noting an increase (92) in positive symptoms.

#### 3.3.4 General psychopathology symptoms

Four studies looked at the impact of stimulation on general psychopathology. Two studies observed significant improvements in general psychopathology for participants in both the active and sham arms using iTBS (82, 83). Studies with only an active rTMS or iTBS stimulation arm found no change in general symptoms (86, 87).

#### 3.3.5 Clinical global impression symptoms

Clinical global impression was examined in three studies. One RCT (*N* = 30) and one pilot study found improvement on clinical global impression post-iTBS or tDCS, respectively (82, 84). A small sample study (*N* = 8) did not find a change in clinical global impression following iTBS (87).

#### 3.3.6 Mood symptoms

Four studies examined the effects of cerebellar stimulation on mood, primarily depressive symptoms. One RCT found that depressive symptoms improved in the active rTMS condition relative to sham (81), although this effect was not significant when accounting for baseline symptoms. In contrast, another RCT found that depressive symptoms similarly improved for both active iTBS and sham (90). Two open-label uncontrolled studies with smaller sample sizes found reductions in depressive symptoms among schizophrenia participants after active rTMS and iTBS stimulation (86, 87). In addition to depressive/sadness features, one study examined the effects of iTBS on several mood states (87). The authors reported increased happiness and alertness from baseline to post-iTBS and at 1-week follow-up; sadness also decreased from baseline to post-stimulation. Other mood ratings (i.e., calmness, wellbeing, anger, self-confidence, fear, and energy) showed no significant differences post-stimulation.

### 3.4 Effects of cerebellar stimulation on cognition and behavior

Six studies examined the effects of cerebellar stimulation on cognition measured from tasks and paper-pencil tests, and three studies examined effects on behavior based on task performance. Three RCTs in individuals with schizophrenia, two of which had relatively larger sample sizes, found no significant effect of iTBS on cognition (82, 90, 93); and that both the active and sham groups similarly improved on multiple cognitive measures over the course of the study (82, 90). In contrast to the null findings for iTBS RCTs, a small open-label study found that patients with schizophenia had improved performance on a continuous performance test and a visuospatial test (87). Gupta et al. (94) also found that individuals with non-clinical psychosis (i.e., high schizotypy) performed better on a pursuit rotor task following active tDCS stimulation; more specifically, the non-clinical psychosis group exhibited a greater rate of improvement on the pursuit rotor task following active stimulation compared to sham, whereas this interaction was not significant for the unaffected comparison group. In fact, the non-clinical psychosis group performed at a comparable level to the control group after active stimulation (94). Additionally, one patient in a case study Laidi et al. (85) improved across a broad range of cognitive functions (i.e., verbal episodic, short term, and working memory, executive, and attention).

Three studies examined how cerebellar stimulation impacted behavior. In a preclinical study, researchers blocked medial prefrontal cortical dopamine receptors in rats as a model of prefrontal abnormalities characteristic of schizophrenia [as evidenced by performance on an interval timing task (95)]. Optogenetic stimulation at 2Hz delta (but not 4, 10, or 20 Hz) of lateral cerebellar projections in these rats rescued behavioral deficits (95). There was no effect of stimulation on other prefrontally-mitigated behaviors, like lever pressing or open-field activity. Comparatively, a clinical study of patients with schizophrenia did not show enhanced performance on the interval timing task following stimulation with iTBS, delta tPCS, or theta tPCS (93). During an eye blink conditioning task which captures associative learning *via* a simple reflex pathway independent of motivation, one individual with schizophrenia showed progressive conditioning after cerebellar tDCS (85).

### 3.5 Effects of cerebellar stimulation on functional brain networks and underlying cortical oscillations

Six studies examined the effects of cerebellar stimulation on underlying brain dynamics, with two studies utilizing resting-state functional connectivity and four studies utilizing electroencephalography (EEG) to derive outcome variables. There is a well-established literature documenting aberrant connectivity between the cerebellum and prefrontal cortex in schizophrenia (96). In a large RCT of individuals with schizophrenia, resting-state functional connectivity increased between the cerebellum and the right inferior frontal gyrus, right pallidum, and right frontal pole following iTBS stimulation relative to sham (90). A different RCT reported increased resting-state functional connectivity between the cerebellum and dorsal prefrontal cortex after active iTBS stimulation relative to sham in participants with schizophrenia (89); further, increased cerebellar-dorsolateral prefrontal cortex connectivity correlated with reductions in PANSS negative symptoms characterized by a large effect size (*r* = −0.81), though we note this sample was quite small (*N* = 11).

Underlying neuro-oscillations measured with EEG can be abnormal in schizophrenia, such as frequencies associated with perception, memory, and synaptic plasticity, including theta and gamma (97–99). Theta oscillatory power was significantly improved following theta tPCS, but not delta tPCS, as evidenced by greater power in the midfrontal region (93). Participants with schizophrenia also showed a more normal pattern of reduced gamma spectral power in the left frontal and temporal cortex after rTMS (86). Further, reduced gamma power in frontal and temporal cortices correlated with negative symptom reductions, while the left frontal cortex corresponded with less severe depressive symptoms (86). In contrast, a case study showed increased gamma spectral power in the left/right frontal and left occipital cortex as well as decreased gamma spectral power in the left temporal region following iTBS (88). In addition to being used as a treatment modality, cerebellar stimulation can be used to probe deficits and to better understand mechanisms underlying the pathophysiology of schizophrenia. In line with this work, Daskalakis et al. (100) used TMS to probe cerebellar inhibititon (i.e., an important measure of cerebellar activity and cerebello-thalamic-cortical pathway integrity) in individuals with schizophrenia. As predicted, individuals with schizophrenia showed significant deficits in cerebellar inhibition compared to unaffected comparison participants.

### 3.6 Effects of cerebellar stimulation on movement

Three studies examined the effects of cerebellar stimulation on movement-related symptoms. Two RCTs found no significant effect of iTBS. In one study, individuals with schizophrenia showed decreased extrapyramidal symptoms and ataxia at 6-week follow-up irrespective of their treatment condition (90). In the other RCT, individuals with treatment-resistant schizophrenia showed no effect of condition or time on extrapyramidal physical symptoms (e.g., gait, rigidity, and tremor) (82). In contrast, a small pilot study showed numerical decreases in clinician-rated tardive dyskinesia (84).

### 3.7 Effects of cerebellar stimulation on physiology

Two studies examined the effects of cerebellar stimulation on physiology (i.e., blood pressure, heart rate/pulse). One RCT found no effect of iTBS; individuals with schizophrenia showed decreased diastolic blood pressure at 6-week follow-up irrespective of their treatment condition (90). An open-label uncontrolled study found that diastolic blood pressure increased immediately post-stimulation and 5 min after, but soon returned to baseline levels. There was no significant change for systolic blood pressure, or heart rate/pulse (87).

### 3.8 Safety/tolerability of cerebellar stimulation

As described earlier, pioneer studies using invasive cerebellar stimulation methods (17–20) had poor tolerability and high rates of non-compliance. Of the 16 studies using non-invasive methods, 10 reported on adverse events and side-effects following stimulation. Of these 10 studies, two reported no side-effects and 6 reported mild side-effects including headaches that were relieved with analgesics (81, 82, 87), pain (83, 87, 90), dizziness and nausea (83), mild skin burn (84), and excessive sleepiness (81). For more serious side effects, cerebellar stimulation (i.e., rTMS) was terminated for one patient due to increased frequency of auditory hallucinations and associated distress (92). Additionally, two participants exhibited increased mania/hypomania after iTBS (90, 91).

### 3.9 Effects in RCTs

When solely focusing on the 7 RCTs that examined cerebellar stimulation in schizophrenia, results remain largely the same as when all studies are included because the results from RCTs (Types 1 and 2) in this systematic review took precedence over less rigorous study designs (Types 3 and 6).

## 4 Discussion

This updated systematic review covers available evidence of cerebellar stimulation effectiveness in treating different symptoms of schizophrenia and influencing underlying neural systems that are deficient in schizophrenia. The number of included studies has more than doubled since the last systematic review (26), and multiple registered clinical trials are in progress. Research designs are becoming more rigorous and sophisticated, with randomized sham-controlled designs, larger samples sizes, and longer follow-up periods. These patterns highlight the increasing attention to cerebellar stimulation as a potential therapeutic intervention and mechanistic probe in schizophrenia.

### 4.1 Clinical symptoms and mood

Over 80% of articles examined whether cerebellar stimulation could alleviate clinical symptoms of schizophrenia. There was some evidence that cerebellar stimulation reduced total psychotic symptoms (i.e., the sum of positive, negative, and general psychopathology symptoms). However, it is unclear whether this reduction was driven by a more specific reduction in negative symptoms. Negative symptoms are thought to account for much of the long-term morbidity, functional impairments, and poor quality of life in schizophrenia, and as such remain a critical unmet need in schizophrenia treatment (101). Cerebellar stimulation was most effective in treating negative symptoms (with some studies reporting reductions maintained up to 24-weeks follow-up), while the findings for positive and general psychopathology symptom reductions were weaker. Because antipsychotic medication is less effective in treating the negative symptoms of schizophrenia (73), the possibility of cerebellar stimulation reducing these symptoms is especially noteworthy. Though we note that existing studies examined overall negative symptoms, and future studies may consider evaluating changes in specific domains of negative symptoms (i.e., experiential/motivational vs. expressive/affective deficits), as they may map onto separate neurobiological systems (102).

Schizophrenia and depression are highly comorbid disorders (103), with both disorders sharing overlapping symptoms, such as anhedonia (104). Initial evidence also raises the possibility that cerebellar stimulation can reduce depressive symptoms in schizophrenia. This is consistent with views of the cerebellum as an “emotional pacemaker,” with the cerebellar vermis in particular believed to modulate emotional processing (105). It is also consistent with research showing that the cerebellum modulates reward processing and controls social behavior (106). Unfortunately, many of the cerebellar stimulation studies that included mood measures lacked a neurostimulation control condition; thus, it cannot be ascertained whether mood changes were the result of active stimulation or simply non-specific treatment effects. However, these studies are an important first step in testing the efficacy of cerebellar stimulation for treating depressive symptoms in schizophrenia.

### 4.2 Cognition and behavior

Surprisingly, cerebellar stimulation did not improve cognition in people with schizophrenia. This contrasts with research in non-psychiatric groups as well as non-psychotic cerebellum-involved disorders, in which participants showed significant gains in learning post-cerebellar stimulation (107, 108). While the previous systematic review (26) concluded that cerebellar stimulation may improve cognitive functioning in schizophrenia, 5 of the 6 papers published after that review showed mixed findings, with comparisons across studies difficult due to study design differences (e.g., different stimulation methods, randomized studies vs. uncontrolled studies), and lack of standardization in cognitive measures and domains assessed. Nonetheless, our understanding of the effects of cerebellar stimulation on cognition in schizophrenia is still an emerging area that would benefit from more rigorous and standardized procedures.

Few studies have looked at whether cerebellar stimulation can impact specific behavioral changes. Interesting findings in rodents showed changes on an interval timing task that captures one’s ability to maintain various temporal intervals in working memory (95); this study raises that possibility that stimulating cerebellar projections to the thalamus may be able to boost cognitive control. Along these lines, another future direction is to examine whether augmentation of this cerebello-thalamic circuit using cerebellar stimulation could modulate sensory prediction deficits present in schizophrenia that depend on this circuit.

### 4.3 Functional brain networks and underlying cortical oscillations

Part of the utility of cerebellar stimulation lies in its potential for having widespread impact on distributed cortical networks (6, 12, 49–52). Consistent with this theory, increased functional connectivity between the cerebellum with the frontal cortex (89, 90) and the right pallidum (90) was observed following cerebellar iTBS relative to sham. Gains in cerebellar-to-prefrontal cortex connectivity were also linked with negative symptom reductions (89), suggesting that modulation of cerebellar-cerebral networks *via* the cerebellum could be an approach to improving symptoms in schizophrenia. Studies examining EEG-related oscillations found that individuals with schizophrenia had a more normal pattern of increased theta oscillatory power in the midfrontal region (93), as well as a more normal pattern of reduced gamma oscillatory power in the left frontal and temporal cortex (86, 88), following tPCS or TMS, respectively. The gamma power reduction corresponded with reductions in negative and depressive symptoms among individuals with schizophrenia (86). Taken together, these studies illustrate how modulation of the cerebellum can impact cerebello-cerebral circuits and their underlying oscillatory dynamics. In turn, this modulation appears to be related to symptom reduction.

### 4.4 Movement

The cerebellum is heavily involved in movement and coordination, and movement abnormalities are present in schizophrenia (109, 110). Based on two RCTs, there was no effect of iTBS on movement-related symptoms (82, 90), although a small pilot study showed numerical decreased in tardive dyskinesia (84). More research in this area is needed to establish the effect of cerebellar stimulation for schizophrenia patients in this domain.

### 4.5 Physiology

The brainstem might be inadvertently affected during cerebellar stimulation, and as such, it is recommended that studies systematically monitor physiological symptoms (14, 21). Of the included studies, two studies examined effects on physiology (i.e., blood pressure, heart rate/pulse) in individuals with schizophrenia. In an RCT, there was no significant effect of iTBS with both active and sham conditions showing decreased diastolic blood pressure at 6-week follow-up (90). An open-label uncontrolled study found increased diastolic blood pressure immediately post-stimulation and 5 min after, with no significant change for systolic either blood pressure or pulse (87). Neither study reported any clinically significant or concerning changes in participants’ physiological activity.

### 4.6 Safety/tolerability

Although the side effect profiles for modern cerebellar stimulation methods are generally low (14, 15), stimulating the cerebellum entails additional risk compared to the rest of the cortex due to its potential to induce painful neck muscle contractions and twitching (14, 21). Across all studies that reported side effects in this systematic review, only two participants reported neck pain during stimulation (that was alleviated with analgesics). Other reported side effects included headaches, dizziness and nausea, mild skin burn, and excessive sleepiness; these side effects were reported in approximately 10% of participants and were mild, temporary, and alleviated by analgesics. Overall, non-invasive brain stimulation methods appear well-tolerated by individuals with schizophrenia and pose minimal safety risks.

### 4.7 Technical issues and considerations when using cerebellar stimulation

To date, the optimal cerebellar stimulation parameters are unknown (15, 21, 44, 111). Efficacy of brain stimulation is determined by coil geometry (for TMS), stimulus intensity, duration and frequency of sessions, depth of the targeted tissue, and location of the cerebellar target. Research in this area is important for increasing efficacy ensuring patient tolerability and developing more personalized treatments.

Despite the cerebellum being a deep brain structure that requires cerebellar-specific stimulation parameters (e.g., coil types and stimulation intensity), most studies have followed standard parameters from cortical stimulation studies (112). Preliminary research on cerebellar-specific stimulation parameters has sought to identify the optimal TMS equipment for effective and tolerable stimulation. These studies compared different TMS coil shapes to find that double-cone (113, 114) and batwing (113) coils, which are designed to stimulate deeper tissue like the cerebellum (115), can effectively stimulate cerebellar targets, with the double-cone-coil being the most effective. Comparatively, one report concluded that the standard figure-of-eight coil produced unreliable results (113). Tolerability of the double-cone coil was significantly less than that of the figure-of-eight, and the authors therefore recommended a double-cone coil at 60% maximal stimulation output to balance reliability and tolerability. Of note, in our systematic review, one study used the angled double-cone coil while the remaining TMS studies used the standard figure-of-eight coil. As for location of the cerebellar target, most of the transcranial electric stimulation studies identified the cerebellar vermis as 1–2 cm below the inion, which is consistent with recommended practice and the majority of cerebellar stimulation studies (15, 33). However, some studies used different methods to identify the cerebellar target, such as MRI-guided neuronavigation (87, 89–91) and the 10–20 international EEG coordinate system (82, 86, 88, 92). This distinction is relevant as neuronavigation helps maximize the precision of the stimulation location for a given individual. Standardization is needed as electrode placement can impact the direction of the current flow direction and orientation of the electric field (15, 33). In line with this, there has been an effort to optimize and standardize procedures of transcranial electric stimulation for cerebellar targets (15, 116). These studies devise a protocol covering optimal electrode montages for cerebellar stimulation, for balancing optimal efficacy with minimal side effects.

Another technical issue to consider when conducting cerebellar stimulation RCTs is selection of the sham condition. There is great variability in sham methods employed in the field (e.g., similar sound and scalp contact but without stimulation, stimulation using the same pulse frequency but with the coil flipped, stimulation using a different frequency, etc.). Selection of the sham condition can lead to differential biological effects beyond the intended transient sensations, which in turn affect the results (117). For sham TMS, changing the position of the coil does not completely exclude residual brain stimulation, which is why one common method is to turn the coil upside down (118). Another recommended approach is to combine a purpose-built coil that mimics sound and scalp contact with surface skin electrodes that provide electrical stimulation time-locked to a TMS pulse (119). For sham tDCS, common approaches are to apply stimulation for a few seconds at the beginning of a session or to stimulate at a constant low intensity for the entire duration (118). It is important to ascertain the efficacy of blinding to condition for both participants and researchers (117), which the majority of the included RCTs did not do. While it has become more common to report on blinding success, this is not yet the standard in the field. Participants’ and researchers’ expectations regarding stimulation/treatment can produce placebo or nocebo effects that impact results (118).

### 4.8 Recommendations and future directions

A major advancement in this field (that is currently underway) is the implementation of RCTs to evaluate cerebellar stimulation in schizophrenia (11, 13). 57% studies included in this review were designed with an active arm only, meaning there was no control condition to determine the specificity of treatment effects. A strength of a recent study is the direct comparison of different cerebellar stimulation approaches [i.e., theta tPCS vs. delta tPCS (93)]. rTMS has been used the most frequently, especially iTBS which has relative advantages over traditional rTMS in that stimulation sessions are shorter, utilize a lower threshold intensity, and exhibit greater long-term excitatory meta-neuroplasticity (27, 28). It is unclear if using rTMS, particularly iTBS, is based on historical practice or if TMS is more effective than transcranial electric stimulation approaches (i.e., tDCS, tPCS, or even transcranial alternating current stimulation [tACS]) when targeting the cerebellum. These latter approaches could be advantageous as they are not known to induce contraction of neck muscles in patients (21). Moreover, these approaches are also less expensive, more portable, and have potential as in-home treatments.

It has also been argued that research linking clinical symptoms to neurobiological measures is hampered by research design obstacles, many of which were present across these studies (120). Notably, larger sample sizes with greater power are needed to establish the reliability of cerebellar stimulation effects. Most studies to date included fewer than 20 individuals with schizophrenia. Alternatively, standardization across sites and studies would allow for the pooling of data. This point is made not only for the stimulation methods/parameters, but also for the assessments, particularly cognitive batteries (as there is more consistency in the clinical symptom inventories used). Furthermore preclinical models of psychosis are needed to test mechanistic hypotheses of cerebellar stimulation.

Longitudinal designs that extend beyond 6 weeks can help clarify the longevity of effects and whether additional doses/boosters are needed. More nuanced longitudinal studies could also help to clarify whether there are individual plateaus in treatment effects, i.e., the subject-specific point after which there are diminishing returns. Available studies varied widely in terms of when they assessed treatment effects. While some studies assessed change throughout the stimulation period, others only compared pre- and post-completion timepoints.

Additional research is needed to understand who will most benefit from cerebellar stimulation (111). Many studies recruited individuals who were treatment-resistant (17–19, 82, 87, 92) or who had at least moderate symptoms (20, 90, 91); however, it is not clear whether these individuals were more likely to benefit from treatment than those with fewer symptoms. Evaluating individuals across the psychosis spectrum can help elucidate whether less symptomatic individuals or those earlier in the illness course can similarly benefit from cerebellar stimulation. For instance, Gupta et al. (94) provided preliminary evidence that cerebellar stimulation improved cognition in non-clinical high schizotypy individuals, whereas this effect was not present in other studies of chronic schizophrenia patients.

Another future direction is to combine cerebellar stimulation with multiple neuroimaging modalities (MRI, EEG) and behavioral tasks to drill down on the underlying circuits impacted by cerebellar stimulation (121). That is, single studies can benefit from the complementary spatial resolution of MRI and the temporal resolution of EEG to clarify how stimulation modulates specific cerebellar-mediated behaviors. One example is the prediction of self-generated stimuli that is feasibly measured using tasks where participants both vocalize brief sounds and listen to playback of themselves (122). The ability to anticipate self-produced auditory stimuli is notably impaired in schizophrenia, as evidenced by deficient suppression of auditory cortical signals measured with EEG and by failures to deactive auditory cortex (122–124). Importantly, this sensory prediction process is supported by an underlying cortico-cerebellar-thalamo-cortico circuit (10). Testing whether stimulation of the cerebellum can augment the underlying cortico-cerebellar-thalamo-cortical circuit and thus improve sensory prediction, is an important and novel future direction.

A caveat regarding the results of this systematic review is the potential for publication bias, especially since many of the older included studies were small open-label or case studies. Although a systematic review was conducted on multiple databases for published articles, studies finding null results might not have been published due to rejection based on small sample size or because the authors did not attempt to publish the results. As studies in the field shift to larger and more rigorous RCTs or longitudinal designs, which can provide more power for detecting effects and can reduce the probability of a Type II error that is more prevalent in small sample studies, the likelihood of publication of null results becomes greater.

## 5 Conclusion

Taken together, cerebellar stimulation shows potential for alleviating negative and depressive symptoms in people with schizophrenia. The mechanism of action underlying cerebellar stimulation may be through modulation of underlying brain systems and oscillatory dynamics, consistent with previous suppositions that targeting the cerebellum can have widespread impact due to its role in distributed cerebellar-cerebral networks. Advancements in cerebellar stimulation have great treatment potential for schizophrenia, although improved standardization across studies is needed to establish the best practices for implementing these approaches and to identify the specific clinical features of schizophrenia that are most responsive to cerebellar stimulation.

## Data availability statement

The original contributions presented in this study are included in the article, further inquiries can be directed to the corresponding author.

## Author contributions

JH was responsible for compiling the initial set of studies for further evaluation. JH and SA were responsible for selecting the final set of included studies and for extracting data from these studies and drafted the initial manuscript. All authors were responsible for the study concept and design, critically reviewed the manuscript, and approved the final version for publication.
